# Exploring Systems That Support Good Clinical Care in Indigenous Primary Health-care Services: A Retrospective Analysis of Longitudinal Systems Assessment Tool Data from High-Improving Services

**DOI:** 10.3389/fpubh.2017.00045

**Published:** 2017-03-24

**Authors:** Cindy Woods, Karen Carlisle, Sarah Larkins, Sandra Claire Thompson, Komla Tsey, Veronica Matthews, Ross Bailie

**Affiliations:** ^1^School of Health, University of New England, Armidale, NSW, Australia; ^2^College of Medicine and Dentistry, James Cook University, Townsville, QLD, Australia; ^3^Anton Breinl Research Centre for Health Systems Strengthening, James Cook University, Townsville, QLD, Australia; ^4^Australian Institute of Tropical Health and Medicine, James Cook University, Townsville, QLD, Australia; ^5^Western Australian Centre for Rural Health, University of Western Australia, Geraldton, WA, Australia; ^6^Cairns Institute and College of Art, Society and Education, James Cook University, Cairns, QLD, Australia; ^7^Menzies School of Health Research, Brisbane, QLD, Australia; ^8^University Centre for Rural Health, Lismore, NSW, Australia

**Keywords:** quality improvement, Indigenous health, primary health services, primary health care, systems improvement

## Abstract

**Background:**

Continuous Quality Improvement is a process for raising the quality of primary health care (PHC) across Indigenous PHC services. In addition to clinical auditing using plan, do, study, and act cycles, engaging staff in a process of reflecting on systems to support quality care is vital. The One21seventy Systems Assessment Tool (SAT) supports staff to assess systems performance in terms of five key components. This study examines quantitative and qualitative SAT data from five high-improving Indigenous PHC services in northern Australia to understand the systems used to support quality care.

**Methods:**

High-improving services selected for the study were determined by calculating quality of care indices for Indigenous health services participating in the Audit and Best Practice in Chronic Disease National Research Partnership. Services that reported continuing high improvement in quality of care delivered across two or more audit tools in three or more audits were selected for the study. Precollected SAT data (from annual team SAT meetings) are presented longitudinally using radar plots for quantitative scores for each component, and content analysis is used to describe strengths and weaknesses of performance in each systems’ component.

**Results:**

High-improving services were able to demonstrate strong processes for assessing system performance and consistent improvement in systems to support quality care across components. Key strengths in the quality support systems included adequate and orientated workforce, appropriate health system supports, and engagement with other organizations and community, while the weaknesses included lack of service infrastructure, recruitment, retention, and support for staff and additional costs. Qualitative data revealed clear voices from health service staff expressing concerns with performance, and subsequent SAT data provided evidence of changes made to address concerns.

**Conclusion:**

Learning from the processes and strengths of high-improving services may be useful as we work with services striving to improve the quality of care provided in other areas.

## Introduction

There are clear disparities in the health of Indigenous and non-Indigenous Australians, with higher ranks of morbidity and mortality among Aboriginal and Torres Strait Islander populations and rural and remote populations (1, 2). High-quality primary health care (PHC) delivered consistently by PHC services is essential (but not alone sufficient) to “close the gap” in Aboriginal and Torres Strait Islander health outcomes (3). Quality PHC in general relates to the degree with which care complies with agreed best practice and is often defined in terms of efficiency, effectiveness, capability, accessibility, safety, appropriateness, continuity, responsiveness, and sustainability (4, 5). However, despite agreed clinical practice guidelines, there is wide variation in delivery of care and processes for evaluating quality of care. Continuous Quality Improvement (CQI) aims to facilitate ongoing improvement in the quality of primary care by using objective information to analyze and improve systems, processes, and outcomes (6). The evidence for the effectiveness of CQI is mixed and context dependent (7, 8); however, studies have shown that in some settings, it can be effective in improving quality of care (9), professional practice (10), and patient outcomes (11), particularly when used over longer periods of time (12).

Modern CQI approaches are increasingly participatory in their methods, support the use of collaborative team-based discussions and have a “customer focus,” which may be more suited to the Indigenous Australian setting (6, 13–15). Building on the Audit and Best Practice in Chronic Disease (ABCD) tools, the One21seventy CQI tools aim to improve the quality and consistency of PHC provided to Aboriginal and Torres Strait Islander people by using clinical audit data to analyze and improve systems, processes, and outcomes (16). The name One21seventy reflects the commitment to increasing life expectancy for Australian Aboriginal and Torres Strait Islander people beyond 1 year in infancy, 21 years in youth, and 70 years in the lifespan (3). The One21seventy CQI process is an annual Plan + Do + Study + Act cycle that uses a number of tools to gather data to facilitate health centers’ planning, goal setting, and implementation of improvements. The One21seventy process include a range of clinical audit tools and a Systems Assessment Tool (SAT). The clinical audit tools are used to collect data to measure the overall adherence to the delivery of guideline-scheduled services to prevent or manage chronic conditions and provide maternal and child health care (Menzies School of Health Care, 2011). As part of the CQI audit cycle, health services are encouraged to conduct a systems assessment to identify strengths and weaknesses in clinical care and health service systems and areas that should be addressed to enhance quality of care. The SAT is an Australian developed scale used to assess the organizational systems of Indigenous PHC services as part of the CQI process. Use of the SAT initially began in 2002–2005 in 12 Northern Territory PHC services. Between 2005 and 2009, use of the SAT expanded to 63 PHC services in four Australian states and territories and continued to expand from then (17).

A SAT process is ideally undertaken by means of a group meeting involving clinical, administrative, and management staff, following the CQI audit. The SAT is based on the Assessment of Chronic Illness Care scale and adapted for use in Indigenous PHC. It was designed to assess systems across multiple areas of care and to be delivered in group setting. Scores are reached by consensus with prompts provided to increase standardization and reproducibility in scoring [(18), see also Table 1]. In some health services, an external facilitator is brought in to assist with the process. The SAT is used by primary health service staff as both a measurement and developmental tool, thereby enabling health service staff to score their health service systems across various domains necessary for effective care: delivery system design, information systems and decision support, self-management support, links with community and other health services, and organizational influence and integration. The SAT allows PHC services to identify priority areas for system improvement and to track variations in systems performance over successive CQI audit cycles. Following the audits and SAT, health service staff are encouraged to undertake collective goal setting and action planning to enhance the quality of evidence-based care to patients over the next 12 months. Governance varies among Australian PHC services, including government-operated services, community-controlled services with Board management, and a combination of both. Systems of governance are ultimately responsible for the implementation of CQI in Indigenous PHC services, whereas PHC services are generally responsible for the planning and conduct of CQI audits and SAT process.

**Table 1:** **Components of the Systems Assessment Tool**.^a^

Components	Items for each component
Delivery system design This component refers to the extent to which the design of the health center’s infrastructure, staffing profile and allocation of roles and responsibilities, client flow, and care processes maximize the potential effectiveness of the center	Team structure and function Clinical leadership Appointments and scheduling Care planning Systematic approach to follow-up Continuity of care Client access/cultural competence Physical infrastructure, supplies, and equipment

Information systems and decision support This component refers to the clinical and other information structures (including structures to support clinical decision-making) and processes to support the planning, delivery, and coordination of care	Maintenance and use of electronic client lists Evidence-based guidelines Specialist–generalist collaborations

Self-management support This component refers to structures and processes that support clients and families to play a major role in maintaining their health, managing their health problems, and achieving safe and healthy environments	Assessment and documentation Self-management education and support, behavior risk reduction, and peer support

Links with community, other health services, and other services This component refers to the extent to which the health center uses external linkages to inform service planning, links clients to outside resources, works out in the community, and contributes to regional planning and resource development	Communication and cooperation on governance and operation of the health center and other community-based organizations and programs Linking health center clients to outside resources Working in the community Communication and cooperation on regional health planning and development of health resources

Organizational influence and integration This component refers to the use of organizational influence to create and support organizational structures and processes that promote safe, high-quality care; and how well all system components are integrated across the center	Organizational commitment Quality improvement strategies Integration of health system components

*^a^Reproduced with the permission from Menzies School of Health Research*.

This study aimed to identify the processes used in systems assessment and the strengths and weaknesses of the systems in place to support the provision of quality client care using quantitative and qualitative SAT data from five consistently high-improving Indigenous PHC services.

## Materials and Methods

### Service Selection

To select high-improving services, we calculated quality of care indices for Indigenous health services participating in the ABCD National Research Partnership. These indices were based on the delivery of scheduled services against the recommended service provision in four audit areas: maternal health, child health, preventive health, and chronic disease (type 2 diabetes). High-improving services were then selected on the basis of continuing high -improvement over at least two of the four audit tools over at least three audits. The method used to calculate the consistent high-improvement category of health services is described in full elsewhere (19).

Six health services met the inclusion criteria of continuous high improvement. Examination of SAT data from the six health services revealed that one service did not record text (qualitative data) to justify SAT scores and was therefore not included in the analysis. Thus our analysis and findings are based on data from five high-improving Indigenous PHC services.

### Study Design

Longitudinal quantitative and qualitative SAT data from five Indigenous PHC services between 2005 and 2014 were analyzed to identify the strengths and weaknesses that supported or constrained the provision of quality health care.

### Data Collection

Pre-existing prospectively collected longitudinal One21seventy SAT data from five high-improving Indigenous PHC services located in northern Australia were analyzed for this study.

Systems Assessment Tool data are recorded for the five main systems components, and items within each component (Table 1) were as follows: delivery systems design (8 items); information systems and decision support (3 items); self-management support (2 items); links with community, other health services, and other services and resources (4 items); and organizational influence and integration (3 items). When each component of the system is assessed in the team meeting, a score from 0 to 11 is allocated to all items within the component, 0–2 for limited or no support, 3–5 for basic support, 6–8 for good support, and 9–11 for fully developed support. An overall score for each component is the average of the item scores. The overall score for each component is presented on a radar plot, displaying the strengths and weaknesses of the systems components.

Although there is some variation in how services conduct a SAT assessment, the process usually involves a facilitated discussion of a mixed group of staff members. They discuss the performance of the service using descriptors against each criterion and then reach consensus on an agreed score for each element. Full detail is available in the SAT tool, coding guide, and facilitator guide (http://www.menzies.edu.au/icms_docs/256788_Systems_Assessment_Tool.pdf).

Health service staff who participate in the systems assessment can also enter free text to justify the score for each item. The five identified consistently high-improving health services each completed between two and five SATs. Two health services completed a combined SAT covering two and three audit tools.

### Study Services

The characteristics of the five health services categorized as consistent high-improvers in this study are described in Table 2, along with a summary of how SAT processes were conducted at these services. Most are government-operated health services located in remote locations with relatively small populations (<1,000 people). One of the services is a community-controlled service, and another service is in a larger regional community located at a community “crossroads.” All health services are located in northern Australia, and four are located in communities with predominantly Indigenous populations.

**Table 2:** **Characteristics of the selected high-improving services**.

Site	State	Governance	Rurality	Population	High improvement in	Conduct of Continuous Quality Improvement (CQI) audits and SAT tools
1	QLD	Government	Remote	≤500	T2DM Maternal	CQI coordinators have conducted the CQI audits each year from 2011 to 2013 In 2014, QLD Health ceased investment in CQI audits The 2015 audits were facilitated by the project team SAT tools: completed by cluster coordinator Goals for improvement are not set, shared, or implemented with local staff

2	QLD	Government	Remote	≤500	T2DM Preventive Child Health	CQI coordinators have conducted the CQI audits each year from 2011 to 2013 In 2014, QLD Health ceased investment in CQI audits The 2015 audits were facilitated by the project team SAT tools: feedback sessions with the cluster coordinator—local staff develop and implement goals for improvement

3	WA	Government/CC partnership	Remote	≥1,000	Maternal T2DM	Senior staff from regional population health unit conducts the audits with support from Menzies SAT tools: based on data from the partnership’s health-care center and conducted by an external facilitator

4	NT	Government	Regional	501–999	Maternal Preventive	Health service manager organizes and conducts the CQI audits with the assistance of all other clinical staff SAT tools: all staff review reports, look at areas needing improvement and set goals Goals for improvement are discussed in meetings (regular agenda item), general observations, shared decisions on goal for improvement

5	NT	Community controlled	Remote	501–999	Preventive Child Health	CQI audits conducted by primary health-care coordinator located at regional health service organization SAT tools: service participates in weekly QI discussions

*QLD, Queensland; WA, Western Australia; NT, Northern Territory; SAT, Systems Assessment Tool*.

### Data Analysis

Quantitative data ranking systems performance for each service is displayed using radar plots. Free text comments associated with these SAT data were analyzed using qualitative content analysis to identify strengths and weaknesses reported over the period of time each PHC service participated in One21seventy audits. Qualitative content analysis is a technique for systematic, replicable text analysis, used to reduce large amounts of text into fewer manageable codes, and to determine the presence of certain concepts within texts (20).

Each item of text justifying the SAT score for each health service, year, and audit tool was analyzed, and concept occurrences were summarized. These concepts were then descriptively coded. Coding categories were based on the stated strengths and weaknesses within each score justification. Identification of concepts allows for conclusions and generalizations to be drawn based on trends indicative of larger ideas (20).

Quantitative data were analyzed using the non-parametric Wilcoxon Signed Rank test to compare the first raw SAT cycle scores of each site with the final raw SAT cycle scores. The Wilcoxon Signed Rank test was selected due to small numbers (scores range from 0 to 11) and the non-normal distribution of data, and the paired nature of the data. Alpha of <0.05 was considered statistically significant.

## Results

Table 3 shows the year and tool for which SATs were undertaken at each health service.

**Table 3:** **Year and tool of Systems Assessment Tool (SAT) completed at each site**.

	2006	2007	2008	2009	2010	2011	2012	2013	2014
**Service 1**									
Maternal health						X	X	X	
T2 diabetes						X	X	X	
**Service 2**									
Maternal health						X	X	X	
Child health						X	X	X	
T2 diabetes						X	X	X	
**Service 3**									
Maternal health			X	X				X	X
T2 diabetes			X	X			X	X	X
**Service 4**									
Maternal health		X				X	X	X	
Preventive health		X	X			X	X	X	
**Service 5**									
Child health		X	X			A	A		
Preventive health	X	X	X	A					

*X, CQI audit and SAT completed*.

*A, CQI audit completed but not SAT*.

**Figure 1:**
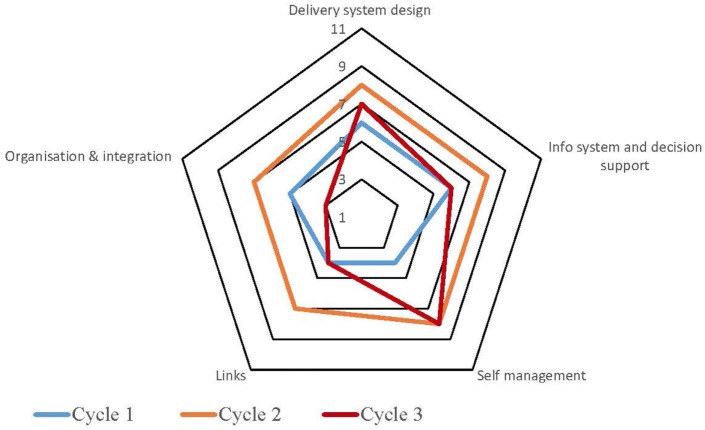
**Service 1: type 2 diabetes and maternal health combined**.

**Figure 2:**
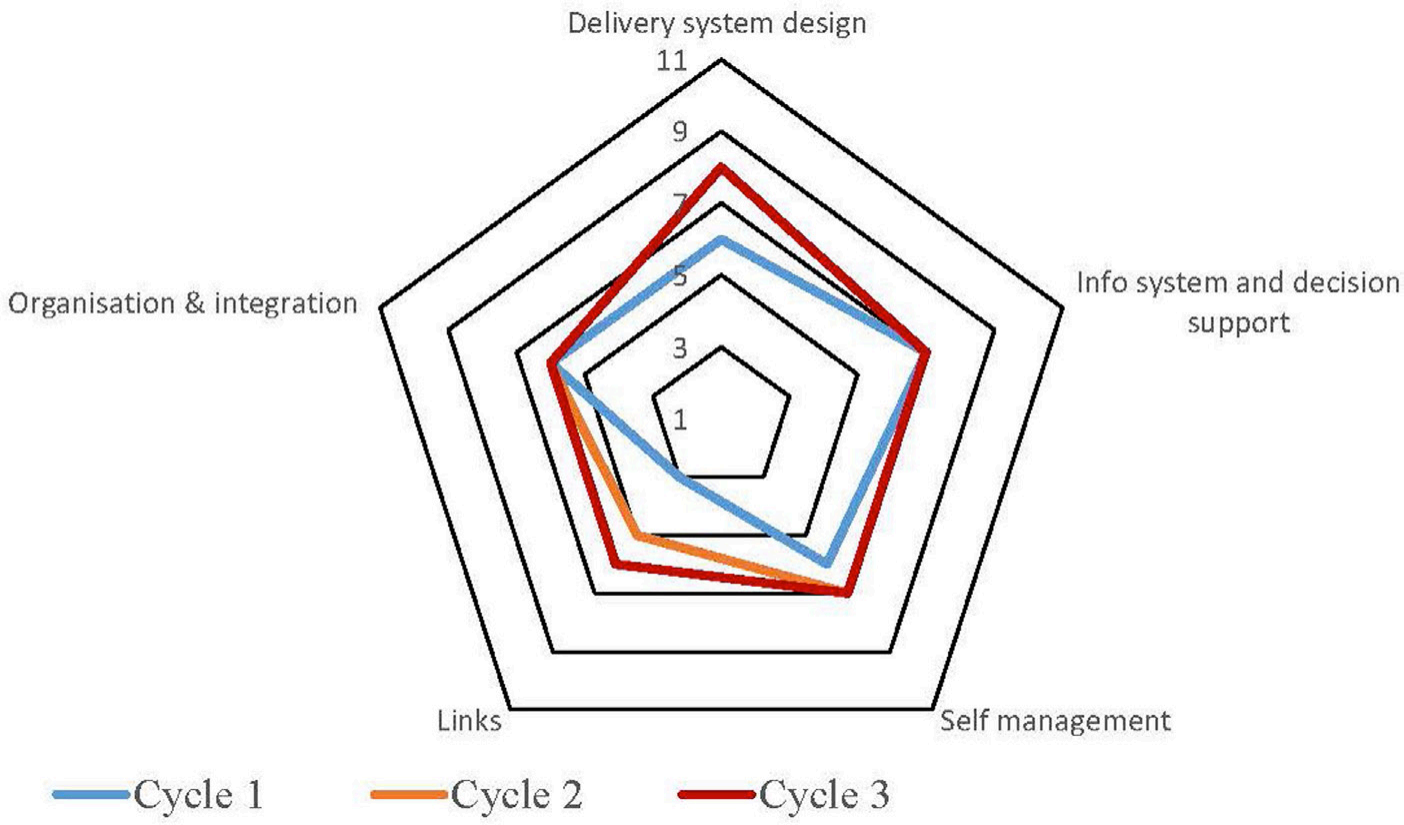
**Service 2: type 2 diabetes, child and preventive health combined**.

**Figure 3:**
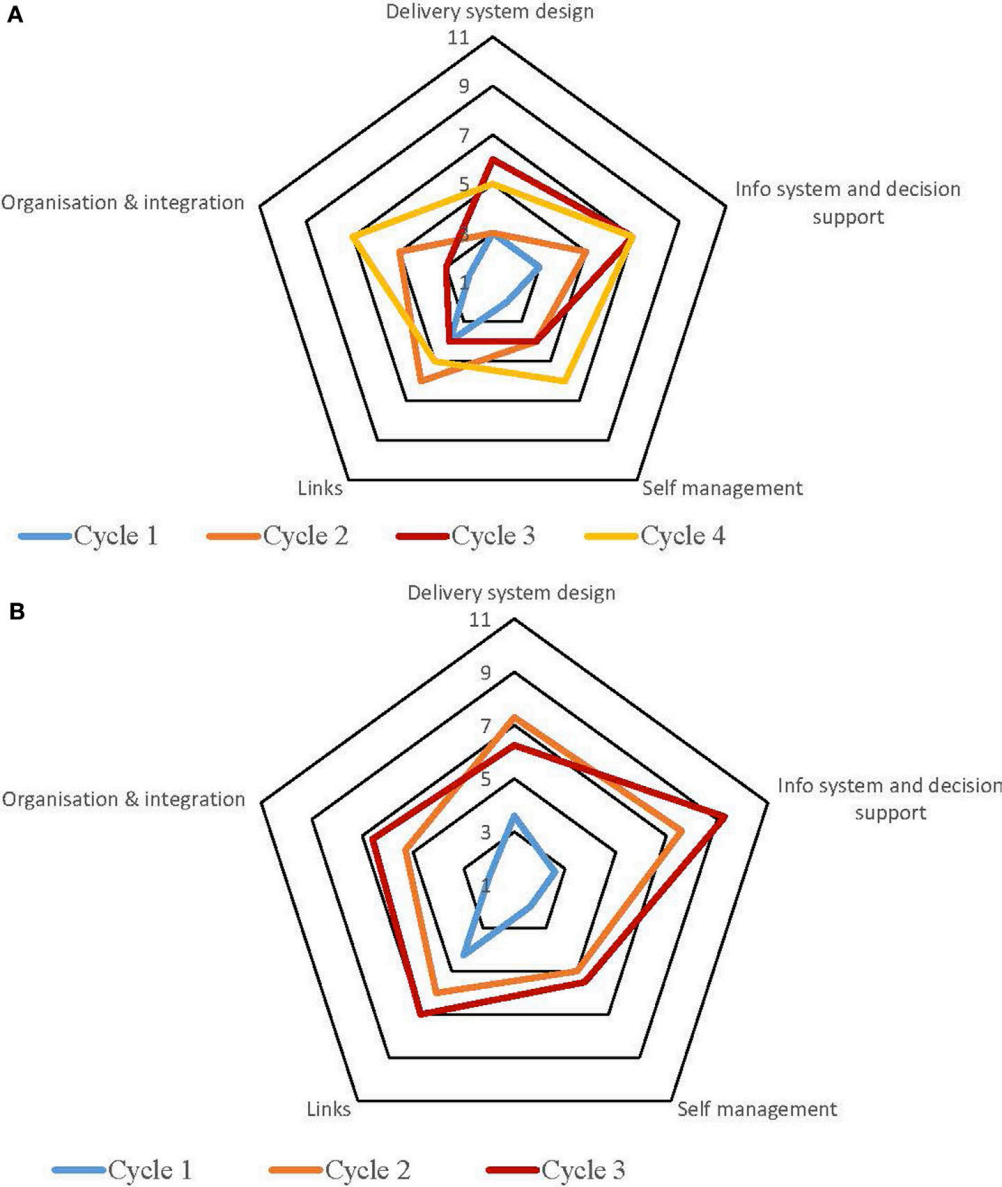
**(A)** Service 3: type 2 diabetes. **(B)** Service 3: maternal health.

**Figure 4:**
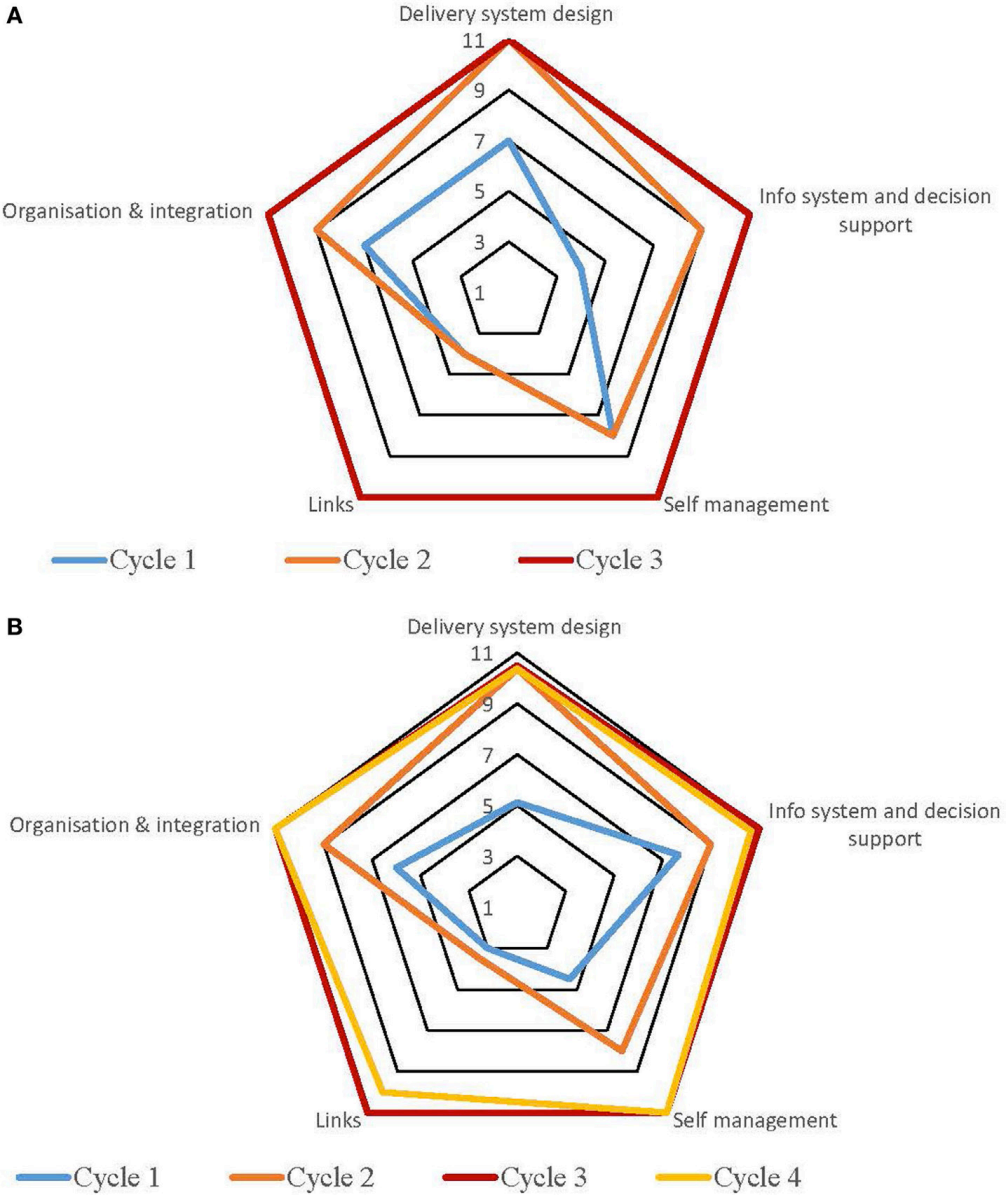
**(A)** Service 4: maternal health. **(B)** Service 4: preventive health.

**Figure 5:**
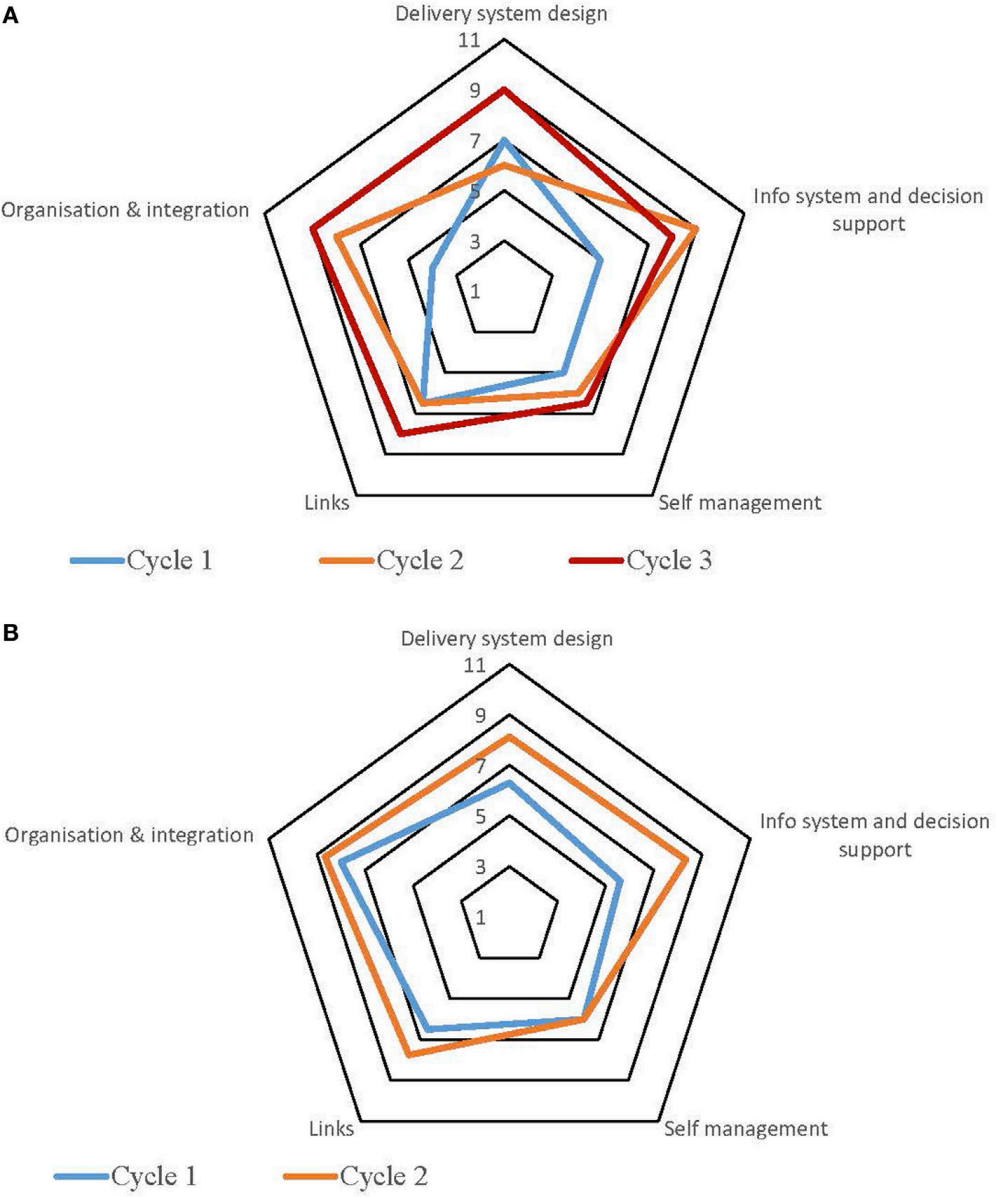
**(A)** Service 5: preventive health. **(B)** Service 5: child health.

Table S1 in Supplementary Material provides a summary of the strengths and weaknesses for each service by each component derived from the free text of the SATs. The process for undertaking CQI audits and completion of SATs varied across the high-improving health services. Some of the services adopted a formal approach which involved all staff members, while in other services, they were carried out by an external team with limited involvement from the health service staff. Figures 1–5 show radar plots demonstrating changes over time in SAT component rankings at each health service. The overall trend showed improvement in each of the SAT components at each service over time; however, as can be seen by the shape of the radar plots, individual services differed in the speed and degree to which various components were addressed.

An overall view of the changes in SAT component rankings over time alongside the reported weaknesses and strengths within each component item are presented in the following paragraphs. Exploration of the justifications for the rankings for each item within each component provides further clarification on why particular rankings were given, illustrate some of the strengths and weakness within the services, and show examples of the impact changes within the service had on rankings of component items within delivery system design. Selected quotations from the free text comments of the SAT process are used to illustrate examples of identified weaknesses and strengths.

### Delivery System Design

Examination of the rankings given across the five services indicated that most of the selected services reported an improvement in this component over time (Figures 1–5). Two services reported improvement from early SAT cycles; however, the most recent SATs showed a contraction (Figures 1 and 3A,B). Key factors related to weaknesses and strengths of delivery system design as identified in the qualitative data were related to recruitment and retention of staff, presence of supportive clinical leadership, appropriate health service systems and processes to support client care and a culturally appropriate and accessible health service. Table 4 shows that sites 3 (T2DM), 4 (maternal health) and 5 (preventive and child health) reported significantly improved delivery system design scores from the first cycle to the final cycle.

**Table 4:** **Mean scores for Systems Assessment Tool cycles**.

Site	Cycle 1	Cycle 2	Cycle 3	Cycle 4	*W* statistic	*P* value
**Site 1: T2DM and maternal health**						
Delivery system design	6	8	7		22	0.34
Info system and decision support	6	8	6		5	0.82
Self-management	4	8	8		–	–
Links	4	7	4		10	0.64
Organization and integration	5	7	3		7	0.37
**Site 2: T2DM, child and preventive health**						
Delivery system design	6	8	8		19	0.18
Info system and decision support	7	6	7		4	1
Self-management	6	7	7		–	–
Links	3	5	6		0	**0.04**
Organization and integration	6	6	6		3	0.65
**Site 3: T2DM**						
Delivery system design	3	3	6	5	9	**0.01**
Info system and decision support	3	5	7	7	0	0.07
Self-management	2	4	4	6	0	0.22
Links	4	6	4	5	3	0.19
Organization and integration	2	5	3	7	0	0.07
**Site 3: maternal health**						
Delivery system design	4	7	0	6	14	0.06
Info system and decision support	3	8	0	9	0	0.07
Self-management	2	5	0	6	0	0.22
Links	4	6	0	7	2	0.10
Organization and integration	2	5	0	7	0	0.07
**Site 4: preventive health**						
Delivery system design	10	10	10		34	0.85
Info system and decision support	9	11	11		4	1
Self-management	8	11	11		0	0.22
Links	4	11	10		0	**0.02**
Organization and integration	9	11	11		0	0.06
**Site 4: maternal health**						
Delivery system design	7	11	11	11	0	**<0.001**
Info system and decision support	4	9	11	11	0	0.07
Self-management	8	8	11	11	0	0.22
Links	4	4	11	10	0	**0.02**
Organization and integration	7	9	11	11	0	0.05
**Site 5: preventive health**						
Delivery system design	7	6	9		11	**0.03**
Info system and decision support	5	9	8		0	0.07
Self-management	5	6	6		1	1
Links	7	7	8		6	0.66
Organization and integration	4	8	9		0	0.07
**Site 5: child health**						
Delivery system design	6	8			13	**0.04**
Info system and decision support	6	8			3	0.81
Self-management	6	6			2	1
Links	7	8			4	0.30
Organization and integration	8	9			2	0.48

*–, indicates numbers are too small to perform statistical test*.

*Bold font indicates statistically significant P values*.

### Team Structure and Function

Qualitative data from early SATs indicated that staffing constraints, recruitment, and retention of staff and issues associated with developing a functional team were key influences on team structure and function. Weaknesses related to staffing included a lack of specialist staff, gender appropriate staff for client groups, appropriate practitioners (particularly Indigenous practitioners) for a team approach, specific staff to oversee programs, and consistent staff. Staffing constraints impacted on the services in different ways. Staff shortages affected training opportunities, regularity of team meetings, the ability of the team leader to coordinate delivery of care, and a lack of a consistent GP affected client follow-up. The following comment highlights the impact of staff shortages on the professional development of key staff, and the Indigenous health worker (IHW) became underutilized.

Trainee HW not being utilised and getting enough training due to no Admin, Trainee is having to do admin duties and they aren’t employed to do admin duties. (Service 1)

In addition, four services reported weaknesses associated with team functioning, including a poorly defined team leadership role, uncertainty and confusion about roles and responsibilities, and irregular team meetings.

Improvement in rankings for this component was associated with adequate staffing levels, thus transforming weaknesses into strengths. When staff issues were resolved, services reported having gender appropriate staff for client groups, practitioners for a team approach, dedicated staff to do recalls, increased training and professional development opportunities, and IHWs were able to fulfill their role and continue their training program. However, many of the improvements were dependent on retention of staff, and when staff left, services were vulnerable. For example, in Service 1, many of the issues with team functioning resurfaced when they lost key staff. Overall, reported improvement in team functioning was associated with clear definition of team roles, team communication and cohesion, and an established team approach for practitioners. Support from management or established reporting mechanisms were also noted as strengths at three services as illustrated below.

Team leadership clearly defined and recognised, leader has formal authority. Definition of team roles, lines of reporting and integration in system design are good. Very good communication and cohesion within the team; team meetings regular; decision-making is very good. Development of team members’ skills and roles is very good. (Service 2)

### Clinical Leadership

Early SAT data in some of the services identified inadequate clinical leadership as limiting the capacity of services to deliver quality health care. Leadership issues varied across the health services and were associated with the availability of leadership staff, tools to support clinical leadership, and the provision of support for on the ground staff. Weaknesses cited included poor medical support for remote health clinics, a lack of clinical leadership, lack of up-to-date evidence-based research, lack of support and direction for staff and programs, and no permanent manager. The comment below from an early SAT process at Service 3 provides an example of some of the factors related to poor clinical leadership.

Poor support and availability from doctors for remote clinics, uncertainty about which clinical guidelines to follow, lack of clinical leadership from GPs (Service 3)

Later SATs showed improvements in clinical leadership. In some cases, leadership staffing issues were resolved or resources were updated to support staff. Strengths such as the presence of strong and supportive leadership, stable management, and access to specialist support were reported as impacting on improved clinical leadership. In other services, innovative solutions were found, for example, comments from Service 3 describe how one midwife extended her skills to carry out her role with high-risk pregnant women, compensating for no on-service support.

The role of the midwife is as a sole practitioner within a community health team but often with no on-site professional skills and support. She either phones or emails the DMO in [nearest regional centre] (250kms away) or the regional obstetrician based in [nearest city] 450 kms away. Therefore the midwife requires extended skills to enable her to carry out her role with high risk pregnant women i.e ultrasound dating scans. (Service 3)

### Appointments and Scheduling

Qualitative data from early SATs indicated that lower rankings for the item appointments and scheduling were due to a lack of an appointment system, staffing to manage appointments, and lack of routine planning for community programs. For some services, inflexibility with their appointments and community programs were described as justification for a lower ranking.

Clients are used to DE [Diabetes Education] clinics on specific days in specific communities… specialist appts are not flexible, surgery list needs to be longer to accommodate clients (Service 3)

Later SATs from three of the services reported that when an appointment system and routine clinics were established, rankings improved. Strengths identified in those settings included, regular clinics, activities and programs, and regular doctor and specialist staff visits.

The [Diabetes Educator] works in specific areas on specific days so people get into a routine…Doctors have routine visits to communities. Specialists and Allied Health have an appointment system. (Service 3)

By contrast, other services found that the communities they worked with expressed a preference for the flexibility of a drop-in service and thus made changes to their systems to meet the needs of the community. One health service noted that “*People know they can come to the health service at any time (service 5)*” indicating there is flexibility built into the system to handle clients without an appointment.

### Care Planning

Care planning processes varied between health services but in most cases improved over time. Early SATs indicated weaknesses in care planning were associated with no electronic medical records or integrated IT system, lack of IT access and training (for remote clinics), no electronic recall system, inaccessibility and inconsistency of care plans, and poor or no documentation.

IT systems did not help with [Adult Health Checks] AHCs which were mostly opportunistic, & generally not completed. Men’s health was not good, and there was a need for a more integrated IT system. (Service 4)

A second issue identified by some services was the capacity of clinicians to undertake planning activities. Qualitative data from the SATs identified issues such as lack of staff to update care plans, doctors not using care plans on IT system, and hospital doctors not seeing the development of care plans as their responsibility.

Improvements in care planning in later SATs were related to improvements in the availability and use of IT systems. For example, the adoption of flags and follow-up reminders used in paper client records, the routine use of the IT system for information sharing and electronic recall lists were reported by services as strengthening care planning. In addition, other reported strengths of care planning included routine use of care plans, a team/specialist approach to complex care, and case conferences with families/specialists. The comment below describes a solution adopted at one health service where there was limited capacity at local level; therefore, support in relation to care planning was provided at the regional board level.

Complex clients’ care incorporates local GP, Maternity Services, [nearest tertiary] hospital, all involved when necessary. (Service 4)

### Systematic Approach to Follow-up

Similar to care planning, client information system weaknesses and staffing constraints were reported in early SATs on the component item systematic approach to follow-up. Issues such as poor actioning of electronic flags/reminders, poor IT access and training, and inconsistent use of IT system for patient records and recalls were some of the reasons cited for lower rankings as part of the SAT process. The comment below describes one example of some of how the health service staff underutilized the IT system.

No use of recall system; C/care [IT system] in remotes; client records multiservice location & not complete; some data sharing between providers; multi-entering of same data. (Service 3)

A review of systematic approach to follow-up in later SATs highlighted improvements to how the IT systems were utilized. Key reported strengths across the services were an increase in the routine use of the IT systems to support follow-up and the availability of dedicated staff.

Flags/reminders consistently used to support client care. Follow-up of clients for regular reviews is becoming part of routine practice. Follow-up of abnormal test results is becoming part of routine practice. (Service 2)

### Continuity of Care

Rankings for continuity of care given by health service staff fluctuated over time for two reasons, staff turnover and poor communication. Poor communication at the point of discharge, from visiting specialists and across IT systems were some of the reasons cited as impacting on continuity of care. The excerpt below highlights how one service identified that its delivery system was not designed to facilitate continuity of care, and steps were being made to address the issue.

The delivery system was not designed to enhance continuity of care, and a system for routine post-discharge communication between hospital [and] the health centre was becoming established (Service 2)

When health services reported improvements in continuity of care, strengths included establishing a system of information sharing, shared care, and shared planning with other health-care providers. A later SAT conducted in Service 2 ranked continuity of care higher than in the previous SAT with the reasoning detailed below.

Very well-designed delivery system enhanced continuity of care (with all or almost all elements in place), and a system for routine post-discharge communication between the hospital and the health centre was fully established. (Service 2)

### Client Access/Cultural Competence

Only one of the five services reported weaknesses in client access. Many of the issues were related to the physical infrastructure of the health services, such as wheelchair access, appropriate waiting room, and consultation rooms. Other issues were related to availability of transport and distance required to travel to attend appointments. The comment below describes an example of lack of an appropriate, private space that created barriers for women who may want to access maternal and child services.

Women have to ask the hospital receptionist to see the midwife or child health nurse. This could be a barrier to access if women are shy having to ask to see a midwife- which has to be said publicly in the waiting area. (Service 3)

In terms of issues related to cultural competence, two of the health services reported weaknesses. Similar to other component items within delivery system design, many of the reported weaknesses were as a result of staff constraints (for example, no or limited IHW) and availability of appropriate training to support staff (for example, cultural competence and gender awareness training).

Conversely, key strengths associated with higher rankings of client access included a clinic designed for client privacy and confidentiality and private consultation areas for men and women. Identified strengths within this component item comprised the availability of cultural orientation and training; in some contexts, this training was provided by Indigenous persons; Indigenous knowledge valued and gender appropriate staff are available for users of the services. The comment below is an example of some of the reasons why cultural competence was ranked highly at one health service.

Level of attention to cultural competence is good; usually included in orientation and training. Respect for gender-related issues is very good. Respect for Indigenous knowledge and IHW experience is very good. (Service 1)

### Physical Infrastructure, Supplies, and Equipment

Data collected as part of early SATs showed that physical infrastructure was reported as a weakness at three of the health services and reasons cited overlap with client access issues reported above. Weaknesses identified included inappropriate and lack of privacy of the waiting area and consultation rooms, lack of disability access, and space constraints for staff, visiting specialists, and clients. The comment below describes the physical infrastructure of one service which impacted on client care.

Clinics unsuitable for client care due to cramped conditions, lack of equipment and no consultation rooms. (Service 3)

Indeed, a more recent SAT process acknowledged that some of the infrastructure issues were resolved with the addition of new buildings and renovation and maintenance of existing buildings.

New clinic, new office, maintenance of equipment is timely, remote clinics being renovated/maintenance. (Service 3)

Health services that ranked physical infrastructure highly reported strengths such as appropriate infrastructure, quality equipment, and systems in place to manage timely maintenance.

### Information Systems and Decision Support

The scoring of this component as shown in the radar plots indicated a mixed picture of rankings over time. Two health services (Figures 3 and 4) showed improvement in the rankings over subsequent cycles. Two of the health services (Figures 1 and 5A) showed an improvement from cycle 1 to cycle 2; however, there was a contraction in the third cycle. One service (Figure 2) reported no change over time. Factors associated with weaknesses and strengths in information systems and decision support were related to the embeddedness of systems, the extent to which they were used to inform planning and support client care and collaboration with other health providers. Table 4 shows increases in information systems and decision support scores are nearing statistical significance at sites 3, 4, and 5. Although changes in scores did not reach statistical significance, it is probable they represent clinical significance in terms of improvement in the quality of planning, client care, and collaboration at these sites.

### Maintenance and Use of Electronic Client List (ECL)

Early SAT data indicated that low rankings of maintenance and use of ECLs were due to a lack of routine use of the client lists and out of date information within the client lists. Those services that experienced a contraction in the rankings in later SATs cited irregular use of the ECLs as the reason for a lower ranking. The comment below is an example from one of the services who assigned a low ranking to this component item.

List available but not reviewed and out of date (covers less than 80% of clients, up-to-date residence and Medicare information sometimes recorded). Use of the list to identify regular clients for planning and delivery is ad hoc. (Service 1)

Services that reported improvements in maintenance and use of client list in later SATs cited strengths such as current and regularly updated ECLs, regular use of recall lists, and use of ECLs for planning and service delivery. One health service described the change in rankings over time as a result of the introduction and routine use of an electronic system.

A barrier of preventive health maintenance and use of electronic client list was the lack of an electronic system. This improved with the introduction of an electronic system although initially it was irregularly reviewed. The electronic client list is used routinely for planning service delivery and reaching client groups and is updated regularly. (Service 4)

### Specialist–Generalist Collaborations

The processes to support the planning, delivery and coordination of care at the PHC level are, to a certain extent, dependent upon collaboration with other health-care providers. Only one of the health services reported weaknesses in collaborations that include no consultation, communication or feedback from specialists, limited specialist visits, and no client access to follow-up after specialist appointments.

Little support from Obstetrician. Gynaecologist visit 5 hrs 3 times a year not enough. No visiting Endocrinologist yet most women diabetic & high risk. Hard to know if specialists recall patients. (Service 3)

The reported weakness in collaboration was resolved in later cycles as a result of engagement in collaborative activities to improve relationships and links with specialists.

Good working relationship with staff and co-location of clinics helps with communication, Paediatrician and Obstetrician contribute to the MCH workshop which helps build relationships. Effective specialist links. (Service 3)

Other health services that ranked specialist-generalist collaborations highly reported similar strengths such as building good relationships, communication, and availability of support.

### Self-management Support

The radar plots (Figures 1–5) show that this component item generally improved over time across all the selected health services. Three services showed continuous improvement over the SAT cycles (Figures 1, 3A,B and 4A,B). Two services showed an improvement from early SATs, which were maintained (Figures 2 and 5). Factors associated with weaknesses and strengths in self-management support included processes and resources to support self-management, availability of appropriately trained staff, and engagement with families and communities.

### Assessment and Documentation

Five services identified weaknesses with assessment and documentation within self-management support in early SAT processes. Processes that supported clients and families to maintain their health were reported with varying success across the services depending on the wider context of the health service. For example, one health service reported that although they were aiming for self-management, it was considered idealistic due to low client health literacy. For other services, high staff workload impacted on the routine use of self-management needs assessment with clients. Furthermore, another health service reported the use of hand-held records as a weakness due to clients declining them.

Don’t always have time to educate, clients not always ready to be educated on self-management. (Service 5)

Improvements in this component item in later cycles were attributed to strengths such as consistent use of self-management needs assessments and ongoing engagement with clients and their family in goal setting and care planning.

Assessment and documentation of self-management needs is routine practice. Clients/families engagement in assessment and documentation is routine practice. (Service 1)

### Self-management Education and Support, Behavior Risk Reduction, and Peer Support

Early SAT data from most of the services identified weaknesses in support structures to help clients and families to manage their health problems. Some issues were related to supporting health service staff such as adequate staffing and staff time, time constraints, and the lack of staff education, training, and skills. Others were related to the ways in which health service staff engaged with the community as illustrated by the comment below.

Ad hoc engagement of families in education/support activities (Service 2)

Qualitative data from later SATs showed that identified weaknesses resolved into strengths when staff had relevant training, skills, and appropriate resources to provide self-management education. In addition, ongoing engagement with families in education/support activities was noted as a reason for improved rankings within this component item. This comment from one of the services describes how changes made within the service—provision of training and embedding processes—improved self-management education and support.

Good self-management education and support by staff with relevant training and skills. Engagement of families in education/support activities becoming routine practice. Use of resources to support self-management becoming routine practice. Behavioural interventions by staff with relevant training and skills becoming part of routine practice. Promotion and support for peer support is becoming central, strategic part of care. (Service 2)

### Links with Community, Other Health Services, and Other Services

Examination of the rankings given across the five services within this component item indicated that most of the selected services reported an improvement over time (Figures 1–5). However, two of the services reported improvements from initial SAT processes, but then, more recent SATs showed a contraction (Figures 1 and 3A). Many of the identified weaknesses and strengths associated with this component item were attributed to the systems in place (or lack of) to facilitate engagement with communities and other service providers. Table 4 shows that sites 2 (T2DM, child and preventive health) and 4 (preventive and maternal health) reported significantly improved links with the community and other services scores from the first cycle to the final cycle.

### Communication and Cooperation on Governance and Operation of the Health Center and Other Community Based Organizations and Programs

The use of community or external linkages to inform service planning varied between services but generally strengthened over time. Weaknesses reported by the health services included a lack of community and client feedback and formal agreements in place for collaboration with other services. This comment from Service 1 is an example of limited communication and cooperation in relation to health service planning and governance.

No community input to governance, no client involvement in planning and feedback, no formal agreements with other services, and client satisfaction rarely assessed. (Service 1)

Over time, all services developed partnerships and communication with other services and community groups. Health services reported strengths such as having formal agreements with organizations, and systematically collecting and using client feedback to inform service planning.

Community input to governance is good. Service population involvement in planning and feedback is becoming systematic. Assessment of client satisfaction becoming systematic and routine. Formal agreements with other services with very good communication and levels of activity. Partnerships with community groups are very good. Health orientation of community programs is very good. (Service 2)

However, some services reported lower rankings of this component item in more recent SATs indicating that more work was required in maintaining the process of engagement with the community and other services.

### Linking Health Center Clients to Outside Resources

The extent that health services linked clients to outside resources was initially low across all services. Weaknesses cited by the health services were related to not having up-to-date referral directories, limited use of the referral directories, and linkages were not well integrated into staff orientation and training. Service 4 cited this component item as an area for improvement and part of their improvement plan.

Included on business plan as an area to improve on. Limited links, some referrals. Some links with QUIT, Healthy Living NT – but no directory present, random, when needed. (Service 4)

Over time services developed a comprehensive, updated and accessible resource directory, and linkages were included in staff orientation and training, and clients were regularly linked to outside resources. The comment below from one health service describes steps made to improve this component item.

Arrangements for linking clients to outside resources becoming systematic, comprehensive resource directory with good updating accessibility and use, and fair integration of linkage arrangements in staff orientation or training. (Service 1)

### Working in the Community

Initially, health service staff work in the community was minimal. Staffing constraints, a high workload, and minimal staff engagement with health promotion and development activities were cited as weaknesses, as illustrated by the comment from Service 3.

Midwife and [health service] staff trained in Core of Life for teenagers but difficult to deliver program due to high workload. (Service 3)

Over time, working in the community became part of most of the health services’ core business. Four of the five health services engaged in community health promotion and development activities, community activities had become integrated into the health service program, and outreach into schools and community education days were occurring. This comment from Service 5 describes some simple steps taken to improve their working in the community.

Staff frequently visit families at home to discuss their kids - all the staff know everyone in the community - go to crèche to talk to mums & kids (Service 5)

### Communication and Cooperation on Regional Health Planning and Development of Health Resources

The extent to which the health services contributed to regional planning and resource development improved over time. Initial SAT data indicated that services had no or minimal engagement in regional health planning or resource development, nor local planning.

No or minimal engagement in regional planning, no or minimal contribution to the development of resources, no or minimal use of community plans (Site 1)

Improvements in rankings for involvement in regional planning were reported in later SATs. Strengths include engagement in regional planning, writing and reviewing regional protocols, representation on regional interagency committees, planning partnerships, and consultation in resource development. Service 3 reported in an early SAT that they were “*only involved in local planning, not regional*”; however, a more recent SAT indicated their involvement had increased.

Strong planning and involvement through partnerships and [Service 3] Futures Forum. Development of contextually appropriate health resources noted. (Service 3)

### Organizational Influence and Integration

Rankings for this component generally indicated improvements over time for most services. One service reported improvement in two of the SATs; however, the most recent SAT showed a contraction (Figure 1). Another service reported no changes to the ranking over time (Figure 2). Qualitative SAT data suggested that weaknesses and strengths of organizational influence and integration were related to adequate funding, appropriate staffing levels, and conditions of work. Table 4 shows increases in organizational influence and integration scores are nearing statistical significance at sites 3–5. Although changes in scores did not reach statistical significance, it is probable that increased scores represent clinical significance in terms of improvement.

### Organizational Commitment

Early SATs indicated that organizational structures and processes that promoted safe, high-quality care were constrained by funding issues and staffing levels. The key weaknesses were reported as staff recruitment and retention, which affected staff workload, training and professional development opportunities, and staff morale.

Staffing levels don’t meet the client’s needs. No specific funding or job description. (Service 4)

With adequate funding and staffing levels, rankings of component item organizational commitment improved. Reasons cited for improvements included manageable workloads, availability of training and professional development opportunities, and communication and staff morale improved.

Plans in place; level of commitment is good. Specific funding, level is fair and/or short term. Level of staffing is good; most roles defined and reflected in job descriptions. Relationships and communication are very good. Morale is very good. Range of training and in-service opportunities is very good. Range of service delivery strategies is good. (Service 2)

### Quality Improvement (QI) Strategies

The key with QI strategies related to participation in QI processes and support. Reported weaknesses in early SATs include issues with the QI process itself, consistent use of QI processes, and limited support from senior staff. The comment from Service 4 is an example of issues raised in terms of preparing staff for CQI and how the CQI process was conducted.

Participation by staff was limited due to lack of training. Staff were expected to review their own processes, the rotating roles within the improvement process was not ideal. Electronic systems were not fully integrated, however incident reporting processes were systematic but no feedback/outcomes. (Service 4)

Later SATs in two health services identified a whole team approach to conducting clinical audits and SATs as a strength. Other strengths included systematic processes for CQI reporting, regular QI education and training, participation in QI collaboratives, and regular assessment of performance against key performance indicators. Examples of activities are provided below.

One21seventy audits and systems assessment undertaken as a team QI activity. (Service 3)

Participate in collaboratives (though time is an issue), ABCD audits, SAT workshops; everyone involved. (Service 5)

### Integration of Health System Components

Key weaknesses of integrated health system components mainly related to staffing and resources issues. At one service, staffing, training, and resource constraints limited the provision of an integrated service.

Recruitment and retention are key issues, IT support from Broome is poor, lengthy delays for new staff to get IT access, and IT access problems at remote sites. (Service 3)

For other services, weaknesses related to IT systems were limitations.

Limited work outside of HC, within community. Information systems not optimal. Business plan reflects the need for partnerships. (Service 4)

Over time, three services reported a good or high standard of integrated service, while a fourth service recognized the importance of integration of service for effective and culturally appropriate care and were working toward this goal.

## Discussion

Evaluation of approaches to CQI is crucial given the rapid growth in the available research on methods of QI and the variability in responses to quality programs (21–23). This paper explored the SAT data from five high-improving Indigenous primary health-care services to understand systems used to support quality care.

Using these data, staffing and support for staff were most commonly reported as influencing the component of delivery systems design. Issues of recruitment and retention also impacted on team work and delivery of quality health care, but these issues resolved over time with adequate staffing levels. Conversely, issues with recruitment and retention of staff led to instances where there was an improvement within this component in one SAT followed by contraction in the following SAT. Leadership and support was limited by remoteness and staffing constraints, and in the absence of adequate staffing or support, innovative solutions were found. An appointment system was viewed as either a strength or a weakness depending on whether clients used the system or preferred a drop-in approach. Scheduling was a strength with regularly planned clinics, activities, programs, and specialist visits. Care planning was constrained by the tools available for planning and the capacity of clinicians to undertake planning activities. Processes in place to support care planning varied across the health services, were dependent on staff availability but in general improved over time. Similarly, a systematic approach to follow-up relied on information systems and adequate staffing levels. Communication between clinicians, visiting staff, the hospital, and other health-care providers was a key strength contributing to continuity of care. The strengths of client access and cultural competence were not only physical access but also cultural awareness, culturally safe practice, and cultural respect. Physical infrastructure impacted negatively on cultural competence when the physical space could not accommodate culturally safe practice.

Within the component of information systems and decision support, information structures such as ECLs, were strengths in the planning and delivery of care when they were current, regularly updated and used as a planning tool. The use of evidence-based guidelines was a strength for clinical decision-making when they were available, accessible, and staff trained in their use. Where information structures and evidence-based guidelines were identified as weaknesses, later SATs revealed changes adopted to ensure they were updated and embedded in practice. Relationships, communication, and support were reported key strengths to coordinate delivery of care with other care providers.

In terms of self-management, identified weaknesses included staff with relevant training, skills, and time to undertake self-management needs assessments, education, and support and to engage with families. Over time, the weaknesses were reported as strengths when staffing issues resolved and training was put in place.

The extent to which the health services used external linkages to inform service planning, linked clients to outside resources, worked out in the community, and contributed to regional planning and resource development varied between services but generally strengthened over time. PHC services developed partnerships and communication with other services and community groups and used client feedback to inform service planning. Working in the community became part of the health services’ core business. PHC services engaged in community health promotion and development activities, and integrated outreach and education into their programs. Engagement in regional planning and resource development increased over time. Staffing and training constraints, systematic reporting processes, and IT issues were weaknesses identified in assessment of organizational influence and an integrated health system. Over time, services were able to resolve these issues and report either a fully integrated health system or progress toward this goal. Two of the services reported weaknesses in later SATs due to external factors impacting on how they worked with other organizations. Evidence from the free text responses indicated a transition period in the setting up of MOUs with other organizations and a review of how feedback from the community was collected were the reasons for the lower scores.

Similar to other component items, inadequate staffing levels and availability of funding were identified as weaknesses in organizational influence and integration. Later SATs showed that when funding and staff levels were addressed, services reported improved ranking of this component.

Our findings indicate that the challenges facing Indigenous PHC services such as lack of service infrastructure, recruitment, retention, and support for staff, and additional costs remain. This is so even in these services selected on the basis of “high-improvement” suggesting a high level of functioning and leadership. The selected high-improving PHC services operate within a complex system responding to different and changing contexts. Despite this complexity, a number of key supportive factors were identified such as adequate and orientated workforce, appropriate health system supports including supportive IT systems and relational factors such as communication and engagement with other organizations and community. Analysis of data collected over time also highlighted the utility of the SAT to help Indigenous PHC services identify areas for change, implement improvements, and monitor those changes over time.

### Strengths and Limitations of the Study

One of the strengths of this study is the availability of longitudinal quantitative and qualitative SAT data from the five selected case study services. Longitudinal quantitative data represented by the scores on the radar plots showed changes in each of the systems components over time. The accompanying qualitative data provided justifications for the SAT scores. The use of both approaches, together with our in-depth knowledge of each service and how QI works from the parallel multiple case studies, makes it possible to capture rich contextual information which in turn can increase understandings of why components are given a particular score. The availability of data over time, showing changes in scoring and justifications why those changes occurred, can allow for some discussion on the degree of amenability to change of each of the important factors. Overall, this increases our knowledge of the extent to which particular factors or conditions can impact on other components of the health system. The inclusion of qualitative and quantitative data from five case study services allows for examination of similarities and differences, which can increase the dependability (24) of the findings.

Our analysis is based on pre-existing SAT data, supplemented by additional information about how the SAT process works. As described earlier, each component of the SAT is assessed in the team meeting and a score is allocated. Health service staff record justifications for the scores as free text. One of the limitations of the data set is that the information was drawn from participating health service staff, providing an incomplete view of the health service; even in those participating, some providers may be more dominant than others. Furthermore, the use of pre-existing SAT data limited the collection of demographic information about the health professionals and their clients involved in the process. The score components (quantitative data) are agreed by a group of staff members from the service with a facilitator. One limitation is that we are unable to correlate their perceptions with actual health system performance retrospectively. In addition, in some services, the SAT process was conducted by an external facilitator and others solely by heath service staff, which may have impacted on how the process was conducted. Generalization of the findings from these five services in northern Australia to other services and contexts must be done cautiously.

### Discussion of the Findings in Relation to Other Relevant Research

Our analysis provides further evidence about the multifaceted contexts within which Indigenous PHC services are operating and significance of these contextual conditions in terms of how they might impact on QI processes. The documentation of rich contextual information allows for a greater understanding of how context and processes might influence QI and goes some way to explaining the variability in responses to particular interventions which may have proved successful in one setting but less successful in another. Two examples reported in our data are (i) difficulties in employing staff and how these impact the provision of health services; or (ii) adaptations made to an appointments system to ensure greater acceptance by the community. Schierhout et al. (23) also identified the complex interaction between context, CQI implementation and variability in responses to CQI. Øvretveit and Gustafson (25) argued that attention to the wider context in implementation of QI interventions “allows exploration of whether and how aligned changes at different levels may result, through complex influences, in better outcomes and how these can be sustained …. this in turn allows decision makers to assess better likely results locally and how to adapt the change” (pi22).

There is general consensus within the literature in terms of the organizational factors influencing successful QI (8, 26). Engels et al. (27) identified five domains of quality in general practice: infrastructure, staff, information, finance, and quality and safety. Similarly, the SAT framework gives due attention to such factors. Our case study data from these same services showed that within the Indigenous PHC service setting, conditions such as adequate staffing levels with strong, supportive clinical leadership in addition to the provision of appropriate orientation, and ongoing training were key strengths for a prepared workforce. Health service system factors were also identified in terms of embeddedness of appropriate, up-to-date and flexible systems to support the planning and delivery of care. Si et al. (21) found that patient-level characteristics contributed substantially to variation in processes of care and suggested that health-care providers need to strengthen their efforts to deliver care and to manage services in a way that most effectively meets the varying needs of individual patients. Our identification of relational factors such as building relationships and regular communication with clients, other health-care providers, and the wider community echoes the review of the literature by Crossland et al. (28), which reported patient-centered approaches, the importance of community, and communication as being integral to high-quality general practice. Furthermore, a report on stakeholder views on strategies for improvement in chronic illness care for Aboriginal and Torres Strait Islander people (29) called for greater partnership working with other health services and more effective links with communities.

Analysis of the SAT data provides evidence of these high-improving health services engaging with the QI process and making changes over time as a result of this engagement. Qualitative data provide valuable insights into reasons why SAT scores changed and the strategies put in place which may have influenced the change. Recent work conducted by Cunningham et al. (18), on the application of SAT data in PHC services, found that respondents reported changes in their health services as a result of using the SAT tool and valued the tool as a lever in implementing improvement. Indeed, Schierhout et al. (23) also found potential causal linkages between CQI activities and outcomes that were achieved. Proposed mechanisms were that the process allowed for identification of issues and prompted change or, alternatively that it, provided evidence and explanations for why things were improving (23).

### Implications of Findings

This study adds to the existing literature on the application of the SAT within an Indigenous PHC setting. The utility of the SAT for CQI is demonstrated through the availability of rich information, which can support service providers in identifying areas of their health system that facilitate QI and increase understandings of how components of the health service interact. Learning from the strengths of high-improving services and identification of what services can do to mediate quality health care may be useful for services striving to improve the quality of care provided in other areas.

## Ethics Statement

This study was carried out in accordance with the recommendations of James Cook University (H6264), Queensland Health (HREC/14/QCH/12-890), Northern Territories Department of Health and Menzies School of Health Research (2014-2299), Western Australian Aboriginal Health Ethics Committee (615), and Western Australia Country Health Service (2015:09) with written informed consent from all subjects. All the subjects gave written informed consent in accordance with the Declaration of Helsinki.

## Author Contributions

The study was conceived and designed by CW, SL and RB. CW, SL, KC, ST, KT, VM and RB contributed to the data collection, analysis, and interpretation; contributed to the synthesis of the findings reported here, critically revised the manuscript for important intellectual content, approved the final version, and agreed to be accountable for all aspects of the work in ensuring that questions related to the accuracy or integrity of any part of the work are appropriately investigated and resolved.

## Conflict of Interest Statement

The authors declare that the research was conducted in the absence of any commercial or financial relationships that could be construed as a potential conflict of interest.
