# Supplementary material for: Exploring Systems That Support Good Clinical Care in Indigenous Primary Health-care Services: A Retrospective Analysis of Longitudinal Systems Assessment Tool Data from High-Improving Services
Source: Front Public Health. 2017 Mar 24;5:45. doi: 10.3389/fpubh.2017.00045 (PMC5364947; doi:10.3389/fpubh.2017.00045)
Supplement: Supplementary file 1 [file table_1.docx]

|  | **Summary of strengths & weaknesses identified from free text responses in SAT data***  Supplementary Materials: Summary of strengths & weaknesses identified in audits for each service | | | | |
| --- | --- | --- | --- | --- | --- |
|  | Service 1 | Service 2 | Service 3 | Service 4 | Service 5 |
| **DELIVERY SYSTEM DESIGN** | | | | | |
| **Identified Strengths** | Increased/regular communication as a team  Adequate staffing  Strong clinical leadership  Introduction of systems for appointments & follow-up  Cultural competence included in training & orientation  Respect for Indigenous knowledge  Good quality & maintenance of physical infrastructure & resources | Team approach with lines of reporting, regular communication & roles  Secured staffing  Recognition of team leadership  Strong clinical leadership  Fully established systems for appointments, care planning & follow up  Good attention to cultural competence, included in orientation & training  Moving towards appropriate & maintained physical infrastructure & resources | Good support from nursing & management  The role of established staff in clinical leadership & up to date evidence based research  Systems in place to remind clients of appointments  Flexibility of appointment systems & introduction of routine clinics  Care plans & continuity of care now routine  Cultural orientation available from Aboriginal service  Improved infrastructure via new clinic space | Availability of up to date evidence based research for staff  Improved systems for appointments & community programs  Whole team approach & sharing of information  System for care planning  Dedicated co-ordinator for preventive health  Continually practice cultural safety with respect for gender & ethnicity | Culture of team work & support amongst staff  Strong, supportive clinical leadership  Very accessible & flexible for clients to attend clinic  Regular follow up & checks  Use of electronic medical records system  Cultural competence routine & attention paid to client privacy & gender |
| **Identified Weaknesses** | Need for regular communication  Staffing constraints  Under utilisation of staff  Team leadership not clearly defined  No appointments system  Ad hoc care planning & systematic approach to follow up  No system for continuity of care | Security of staff roles  Team leadership not clearly defined  Staffing constraints  Ad hoc continuity of care  Physical infrastructure poor & limited availability of resources | Lack of staff, clinical leaders & clarity around roles  Poor accessibility for remote clients  Inadequate orientation, support & training for staff  Care planning & continuity of care ad hoc  Access & privacy issues with infrastructure (limited space) | Staffing constraints  Poorly defined leadership role  Lack of evidence based research  Lack of community programs in place  Minimal participation in community programs  IT systems -Appointment system & care planning system to be established  Limited space for consultations | No-one overseeing programs  Care planning growth action plans needed  No leader for child health program  Follow up & continuity of care not routine (time constraints)  Lack of appropriate equipment |
| **INFORMATION SYSTEMS & DECISION SUPPORT** | | | | | |
| **Identified strengths** | Routine use of electronic client list  Routine use of evidence based resources & training for staff  Good specialist-generalist collaborations | Routine use of electronic client list & implementation of strategies to reach specific groups  Good use of evidence based resources & training for staff in use | Maintenance of electronic client lists  Strategies introduced to improve use of evidence based resources Training & orientations, joint meetings & projects  Good collaboration with good relationships | Introduction of electronic client list  Improved access to updated research & evidence-based guidelines  Good collaboration & specialist support | Good local knowledge & can provide updated information to electronic client list  New staff orientation & all staff use of evidence based guidelines  Significant improvement on specialist-generalist collaboration as a result of improved communication & co-ordinators |
| **Identified weaknesses** | Electronic client list are out of date & irregularly reviewed,  Ad hoc use of list for planning & delivery  Minimal staff training on use of evidence based resources | Electronic client list irregularly reviewed  Minimal specialist-generalist collaboration | Limited support from specialists | No electronic system  No training or availability of all evidence based guidelines | Long wait time for referral appointments |
| **SELF MANAGEMENT SUPPORT** | | | | | |
| **Identified strengths** | Support for self-management, routine assessment & documentation of needs  Routine engagement with clients/families  Good self-management education & support  Outreach, promotion & peer support central to care | Good support for self-management, routine assessment & documentation of needs  Routine engagement with clients/families  Staff with training routinely providing good self-management education  Outreach, promotion & peer support central to care | Project to produce/adapt culturally appropriate resources  Training available for staff in self-management & health promotion | Good use of assessment & documentation to support planning  Routine engagement with family/client  Good self-management, education & support due to educational materials, family involvement, brief interventions & visits | Families involved in care planning  Good health promotion resources & family involvement in self-management education |
| **Identified weaknesses** | Self-management, assessment & documentation of needs not routine  Limited self-management education & staff training | self-management education & support, behaviour risk reduction & peer support are ad hoc  Low use of resources to support self-management | Clients decline h& held records  Limited support for clients to self-manage  Further training & education required | Time constraints & limited resources | Growth action plans not ideal  More health promotion resources  More preventative strategies needed |
| **LINKS WITH COMMUNITY, OTHER HEALTH SERVICES & OTHER SERVICES** | | | | | |
| **Identified strengths** | Staff training in linking arrangements  Working in the community, integration of community activities & newsletter | Systematic community input into governance, planning & feedback  Formal agreements with other services  Availability of comprehensive resource directory & staff training  Integration of community activities into PHC programs | Regular communication to ensure other service input into planning & feedback  Partnership with Aboriginal service to deliver community programs  Regular updates of resource directory for clients  Staff highly involved in working in the community | Some communication with other services & client feedback forms  Some partnerships with outside organisations  Linking clients with outside resources identified as a priority  Working in the community through health checks & recalls  Communication in health planning with outside services | Good links & cooperation with community & other services  Health workers are committee members for community organisations  Stable staff aware of how to access resources  Up to date list of resources available for staff  Staff know all the community members  Active involvement in health planning |
| **Identified weaknesses** | No client involvement in planning & feedback  No formal agreements with other services  No resource directory for staff  No engagement in regional planning | No service population involvement in planning & feedback  Assessment of client satisfaction is ad hoc  Linking health service clients to outside resources is ad hoc  Minimal staff involvement in community health promotion  Fair engagement in regional planning | No community input into health  No MOU between local service providers  Difficulty delivering community programs due to high workload | No system to engage with community  Limited links with other organisations  Limited community programs | Orientation for new staff required  Need to expand community program |
| **ORGANISATIONAL INFLUENCE & INTEGRATION** | | | | | |
| **Identified strengths** | Plans in place for funding & staff training  Good range of service delivery strategies, good relationships & morale | Plans in place for funding, staff training & in-service support  Good range of service delivery strategies, good relationships & morale  Good level of integration of health system components | In-service training  Good systems/strategies in place to achieve CQI  Recognition of the importance of integration of health system components for effective & culturally appropriate care | Plans in place, funding & up to date training  Improvement in CQI strategies & supported by senior staff  Improvement to a fully integrated health system | Co-ordinator in place to provide advice & staff training  -All staff participate in CQI & external CQI activities across health services in the region  External CQI activities a key enabler for integration of health system components |
| **Identified weaknesses** | No plans in place  No funding  Fair staffing levels  Some roles defined  Inconsistent QI support from senior staff | No plans in place  No funding  Fair staffing levels  Some roles defined  Inconsistent QI support from senior staff | Staffing, training & funding constraints  Low morale  More support from senior staff required | Job description not matching specific roles,  Senior staff lack understanding of staff needs  Lack of training in QI for staff | No program lead  Time constraints  More QI training |
